# Discordant congenital Zika syndrome twins show differential in vitro viral susceptibility of neural progenitor cells

**DOI:** 10.1038/s41467-017-02790-9

**Published:** 2018-02-02

**Authors:** Luiz Carlos Caires-Júnior, Ernesto Goulart, Uirá Souto Melo, Bruno Henrique Silva Araujo, Lucas Alvizi, Alessandra Soares-Schanoski, Danyllo Felipe de Oliveira, Gerson Shigeru Kobayashi, Karina Griesi-Oliveira, Camila Manso Musso, Murilo Sena Amaral, Lucas Ferreira daSilva, Renato Mancini Astray, Sandra Fernanda Suárez-Patiño, Daniella Cristina Ventini, Sérgio Gomes da Silva, Guilherme Lopes Yamamoto, Suzana Ezquina, Michel Satya Naslavsky, Kayque Alves Telles-Silva, Karina Weinmann, Vanessa van der Linden, Helio van der Linden, João Ricardo Mendes de Oliveira, Nivia Maria Rodrigues Arrais, Adriana Melo, Thalita Figueiredo, Silvana Santos, Joanna Goes Castro Meira, Saulo Duarte Passos, Roque Pacheco de Almeida, Ana Jovina Barreto Bispo, Esper Abrão Cavalheiro, Jorge Kalil, Edécio Cunha-Neto, Helder Nakaya, Robert Andreata-Santos, Luis Carlos de Souza Ferreira, Sergio Verjovski-Almeida, Paulo Lee Ho, Maria Rita Passos-Bueno, Mayana Zatz

**Affiliations:** 1 Department of Genetics and Evolutionary Biology, Human Genome and Stem Cell Research Center, Biosciences Institute, University of São Paulo (USP), São Paulo – SP, 05508-900 Brazil; 2 Brazilian Biosciences National Laboratory (LNBio), Brazilian Center for Research in Energy and Materials (CNPEM), Campinas – SP, 13083-970 Brazil; 3 Neuroscience laboratory, Department of Neurology and Neurosurgery, Federal University of São Paulo—UNIFESP/EPM, São Paulo – SP, 04039-002 Brazil; 4 Butantan Institute, São Paulo – SP, 05503-900 Brazil; 5 Albert Einstein Hospital, São Paulo – SP, 05652-900 Brazil; 6 Department of Biochemistry, Institute of Chemistry, University of São Paulo (USP), São Paulo – SP, 05508-900 Brazil; 7 Universidade de Mogi das Cruzes, Mogi das Cruzes – SP, 08780-911 Brazil; 8 AACD, Recife – PE, 50080-810 Brazil; 9 Rehabilitation Center—Dr. Henrique Santillo, Goiânia – GO, 74653-230 Brazil; 10 Neuropsychiatry Department and KeizoAsami Laboratory, Federal University of Pernambuco (UFPE), Recife – PE, 50670-901 Brazil; 11 Department of Pediatrics, Federal University of Rio Grande do Norte (UFRN), Natal– RN, 59010-180 Brazil; 12 ISEA, Campina Grande– PB, 58400-220 Brazil; 13 Department of Biology, Paraíba State University (UEPB), Campina Grande – PB, 58429-500 Brazil; 14 Federal University of Bahia (UFBA), Salvador – BA, 40170-115 Brazil; 15 Infectious pediatric laboratory, Medicine School of Jundiaí, Jundiaí – SP, 13202-550 Brazil; 16 Division of Immunology and Molecular Biology Laboratory, Federal University of Sergipe (UFS), Aracaju – SP, 49100-000 Brazil; 17 Heart Institute, Faculty of Medicine, University of São Paulo (USP), São Paulo – SP, 05403-900 Brazil; 18 Department of Clinical and Toxicological Analyses, School of Pharmaceutical Sciences, University of São Paulo (USP), São Paulo – SP, 05508-900 Brazil; 19 Vaccine Development Laboratory, Department of Microbiology, Institute of Biomedical Science, University of São Paulo (USP), São Paulo – SP, 05508-900 Brazil

## Abstract

Congenital Zika syndrome (CZS) causes early brain development impairment by affecting neural progenitor cells (NPCs). Here, we analyze NPCs from three pairs of dizygotic twins discordant for CZS. We compare by RNA-Seq the NPCs derived from CZS-affected and CZS-unaffected twins. Prior to Zika virus (ZIKV) infection the NPCs from CZS babies show a significantly different gene expression signature of mTOR and Wnt pathway regulators, key to a neurodevelopmental program. Following ZIKV in vitro infection, cells from affected individuals have significantly higher ZIKV replication and reduced cell growth. Whole-exome analysis in 18 affected CZS babies as compared to 5 unaffected twins and 609 controls excludes a monogenic model to explain resistance or increased susceptibility to CZS development. Overall, our results indicate that CZS is not a stochastic event and depends on NPC intrinsic susceptibility, possibly related to oligogenic and/or epigenetic mechanisms.

## Abstract (web summary)

Zika virus (ZIKV) infection can cause congenital Zika syndrome (CZS), but the underlying mechanisms are poorly understood. Here, the authors generate neural progenitor cells from dizygotic twins with a discordant phenotype regarding CZS and study their response to ZIKV infection.

## Introduction

Zika virus (ZIKV) is a flavivirus that has been associated with severe brain abnormalities in newborns^1–6^. Neurodevelopmental dysfunctions of congenital Zika syndrome (CZS) was shown to be caused by impairments in neural progenitor cell (NPC) growth and survival^7, 8^. CZS, characterized by microcephaly and other abnormalities (visual defects, hearing impairment, skeletal deformities, and epilepsy), occurs in 6–12% of cases of pregnant women infected by ZIKV^4–6^. These observations suggest that ZIKV infection during pregnancy is not deterministic for CZS phenotype and other susceptibility factors might be involved. In a previous study, McGrath et al.^9^ demonstrated that NPCs from different individuals could respond differently under ZIKV infection. This was observed by differential modulation of intracellular signaling pathways, especially related to innate immunity, cell cycling, and mammalian target of rapamycin (mTOR) signaling.

Discordant twins represent a good case–control sample to test for the genetic contribution determining the fetuses’ outcome of gestational infection. To the best of our knowledge, no other study has compared the in vitro NPC expression profile or outcome of ZIKV infection in human induced pluripotent stem cell (hiPSC)-derived NPCs from discordant twins for CZS in the same experimental conditions. Here we show that ZIKV replicates significantly more in hiPSC-derived NPCs from affected (CZS) babies than in the non-affected counterparts. In addition, transcriptome profiling revealed a different pattern in NPCs from CZS-affected as compared to CZS-non-affected individuals highlighting the role of Wnt and mTOR signaling in modulating ZIKV infection outcome.

## Results

### Subject and samples

**Fig. 1:**
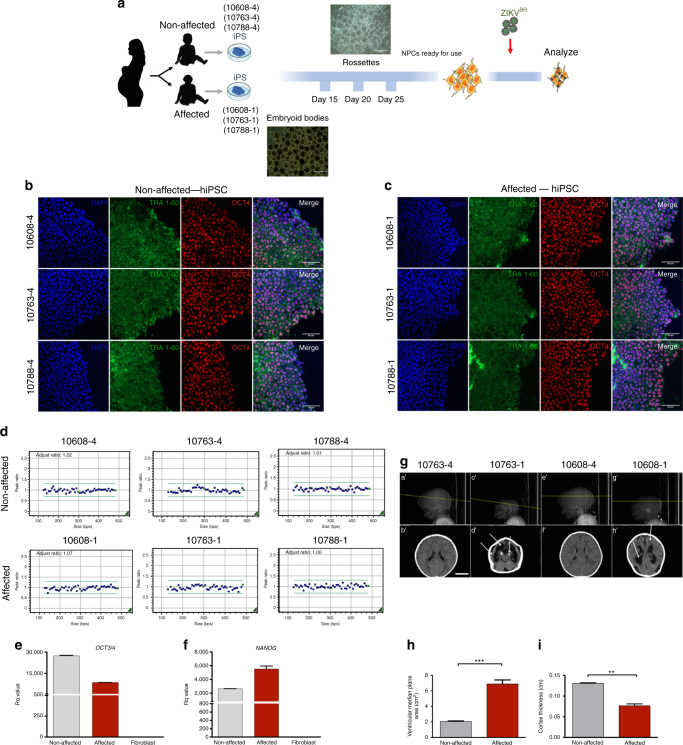
Experiment design and hiPSC characterization. **a** Schematic: generation of neural progenitor cells (NPCs) from discordant twins’ hiPSCs following ZIKV^BR^ infection and analysis. Silhouettes are courtesy of www.vecteezy.com (mother) and Yulia Ryabokon (babies). **b**, **c** Immunofluorescence for TRA-1-60 and OCT3/4 in hiPSCs. Scale bar, 20 μm. **d** MLPA analysis of subtelomeric imbalance chromosomal abnormalities in cultured hiPSCs cell lines using P070 and P036 MLPA Kits. **e**,** f** RT-qPCR analysis of hiPSCs for *OCT3/4* and *NANOG* expression (mean ± SEM). **g** Representative discordant twins’ computed tomography (CT) scan images (a′, b′ [#10763-4]; e′, f′ [#10608-4]—non-affected babies; c′, d′ [#10763-1], g′, h′ [#10608-1]—affected babies). #10608 twins were 5 months old and #10763 were 15 months old when submitted to CT scan. White arrows show observed brain abnormalities (calcifications, ventriculomegaly, and cortical gyrus simplification). Scale bar, 3 cm. **h** Ventricular area analysis (cm^2^) (*n* = 4, two pairs of discordant twins). **i** Cortex thickness analysis (*n* = 4, two pairs of discordant twins). ***p < 0.001; **p < 0.01; mean ± SEM; Student’s *t* test

A total of nine pairs of Brazilian twins exposed to ZIKV during pregnancy were identified: seven dizygotic (DZ) and two monozygotic (MZ). The two MZ twins were both affected (concordant), while among DZ twins, six were discordant (one affected and one healthy) and only one was concordant. Despite the relative small sample size, the rate of discordance among DZ twins and the higher concordance in MZ than DZ twins could suggest the existence of susceptibility factors increasing the risk for CZS. We obtained saliva samples from eight pairs of twins for DNA exome sequencing: two MZ concordant pairs of twins (#10789 and #10835) and six DZ pairs of twins, one concordant (#11113) and five discordant (#10608, #10658, #10691, #10763, and #10788). In addition, DNA samples from 10 unrelated CZS babies were included in this analysis. Furthermore, we obtained blood from three of the pairs of DZ discordant twins for generation of hiPSC-derived NPCs (#10608, #10763, and #10788) (Fig. 1a). All patients’ clinical information is detailed in Supplementary Data 1.

### Whole-exome sequencing analysis

We first analyzed variants in genes related to Mendelian inherited microcephaly or syndromes associated with microcephaly (Supplementary Data 2). We did not identify any pathogenic or probably pathogenic variant in such genes. A larger number of rare variants (minor allele frequency <0.01) in eight genes (*DEPDC5*, *GPR108*, *MICAL3*, *OR12D2*, *OR4K5*, *PHF2*, *SLC6A18*, and *TTC16*) was found in affected as compared to normal babies or control aged population. However, these differences did not achieve statistical significance (*p* > 0.05) after Bonferroni correction. The statistical power was sufficient to exclude monogenic dominant inheritance (99.57%), whereas recessive (31.07%) or multifactorial inheritances could not be excluded as responsible for an increased CZS susceptibility.

### Generation and characterization of hiPSCs

Erythroblasts from three pairs of DZ twins (non-affected: #10608-4, #10763-4, and #10788-4; affected: #10608-1, #10763-1, and #10788-1) were reprogrammed towards hiPSCs. All hiPSC lines expressed markers of pluripotency including TRA-1-60 and OCT4 as demonstrated by immunofluorescence staining (Fig. 1b, c). Cell lines were screened with two Multiplex Ligation-dependent Probe Amplification (MLPA) Kits (P036 and P070, MRC-Holland, Amsterdam, The Netherlands) for subtelomeric imbalances and no chromosomal abnormalities were observed (Fig. 1d). Quantitative analysis (real-time quantitative PCR (RT-qPCR)) demonstrated expression in the generated hiPSCs of selected endogenous pluripotent transcription factors including *NANOG* and *OCT4* (Fig. 1e, f).

### Computed tomography

Computed tomography scan (CT scan) from two pairs of twins out of the three DZ discordant pairs (#10608 and #10763 twins) revealed typical abnormalities associated with CZS in the affected babies, while non-affected correspondent siblings have no visible alterations (Fig. 1g, sub-panels a′, b′, e′, f′). Main findings observed in CT scan of affected individuals were brain calcifications, which were mostly punctiform (Fig. 1g, sub-panels d′, h′). Microcephaly and malformations of cortical development can be seen at Fig. 1g, sub-panels c′, g′ as well as at Fig. 1i. Other findings were ventriculomegaly (Fig. 1g, sub-panels d′, h′, Fig. 1h) and cortical gyrus simplification.

### Infection with ZIKV^BR^ of NPCs from discordant twins

**Fig. 2:**
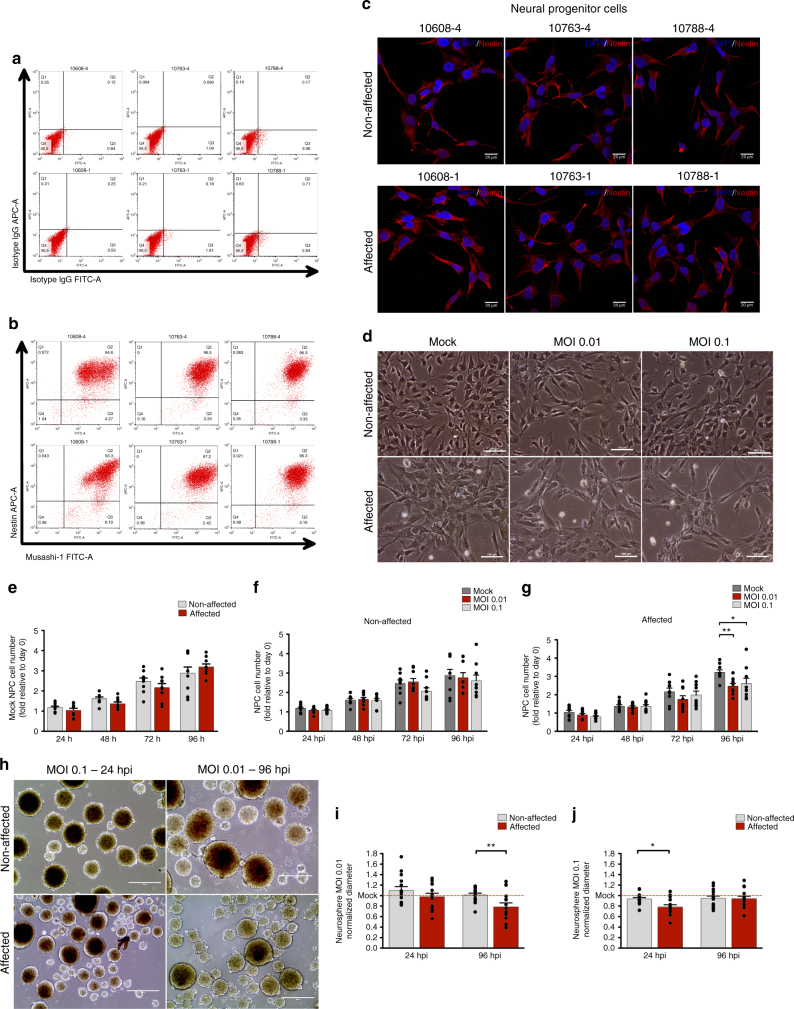
NPC characterization and ZIKV^BR^ infection in DZ-D cells (see also Supplementary Fig. 1). **a**, **b** NPCs were stained with isotype IgG APC-A and isotype IgG FITC-A (**a**) and Nestin APC-A and Musashi-1 FITC-A (**b**) and were analyzed by flow cytometry to confirm (or show) population homogeneity for neural progenitor markers. **c** Nestin staining by immunofluorescence. Scale bar (SB), 20 μm **d** Representative phase-contrast micrograph of infected NPCs (#10608 twins). SB, 100 μm. **e** Analysis of cell number of Mock-infected NPCs from non-affected and CZS-affected twins (*n* = 3 technical replicates; mean ± SEM). **f**, **g** MOI 0.01 and 0.1 infected NPCs from non-affected (#10608-4, #10763-4, and #10788-4) (**f**) and CZS-affected (#10608-1, #10763-1, and #10788-1) (**g**) twins in monolayer cultures at 24, 48, 72, and 96 hpi (*n* = 3 technical replicates; mean ± SEM; *p < 0.05, **p < 0.01; two-way ANOVA with Bonferroni post hoc analysis). **h** Representative images of neurospheres infected with ZIKV^BR^ (#10608 twins, *n* = 2 technical replicates). SB, 400 μm. **i**, **j** Normalized diameter of neurospheres at 24 and 96 hpi with MOI 0.01 (**i**) or MOI 0.1 (**j**); #10608, #10763 and #10788 twins; *n* ≅ 15 technical replicates; mean ± SEM; *p < 0.05, **p < 0.01 Student’s *t* test

Firstly, hiPSC-derived NPCs from three pairs of DZ discordant twins (non-affected: #10608-4, #10763-4, and #10788-4; affected: #10608-1, #10763-1, and #10788-1) were characterized to confirm their differentiation in vitro, using our group’s established protocol for NPC differentiation^10^. Under these conditions, we found that all hiPSC-derived NPC lines robustly and homogeneously express Musashi-1 and Nestin (Fig. 2a–c). Subsequently, we infected hiPSC-derived NPCs with a Brazilian ZIKV (ZIKV^BR^) strain using low multiplicity of infection (MOI) (0.01 and 0.1) (Fig. 2d). A marked reduction of growth was observed in the CZS-affected twins’ NPCs at 96 h post infection (hpi) (MOI 0.01, *p* < 0.01; MOI 0.1, *p* < 0.05; two-way analysis of variance (ANOVA) with Bonferroni post hoc analysis) in monolayer culture (Fig. 2f, g, Supplementary Fig. 1) and no differences were observed at mock conditions among affected and non-affected samples (Fig. 2e). The differences were also confirmed in neurospheres– three-dimensional (3D) cultures (Fig. 2h) with reduction of diameter at 24 hpi (MOI 0.1; *p* value = 0.0045; Student’s *t* test) (Fig. 2j) and 96 hpi (MOI 0.01; *p* value = 0.0116; Student’s *t* test) (Fig. 2i).

### ZIKV infection analysis

**Fig. 3:**
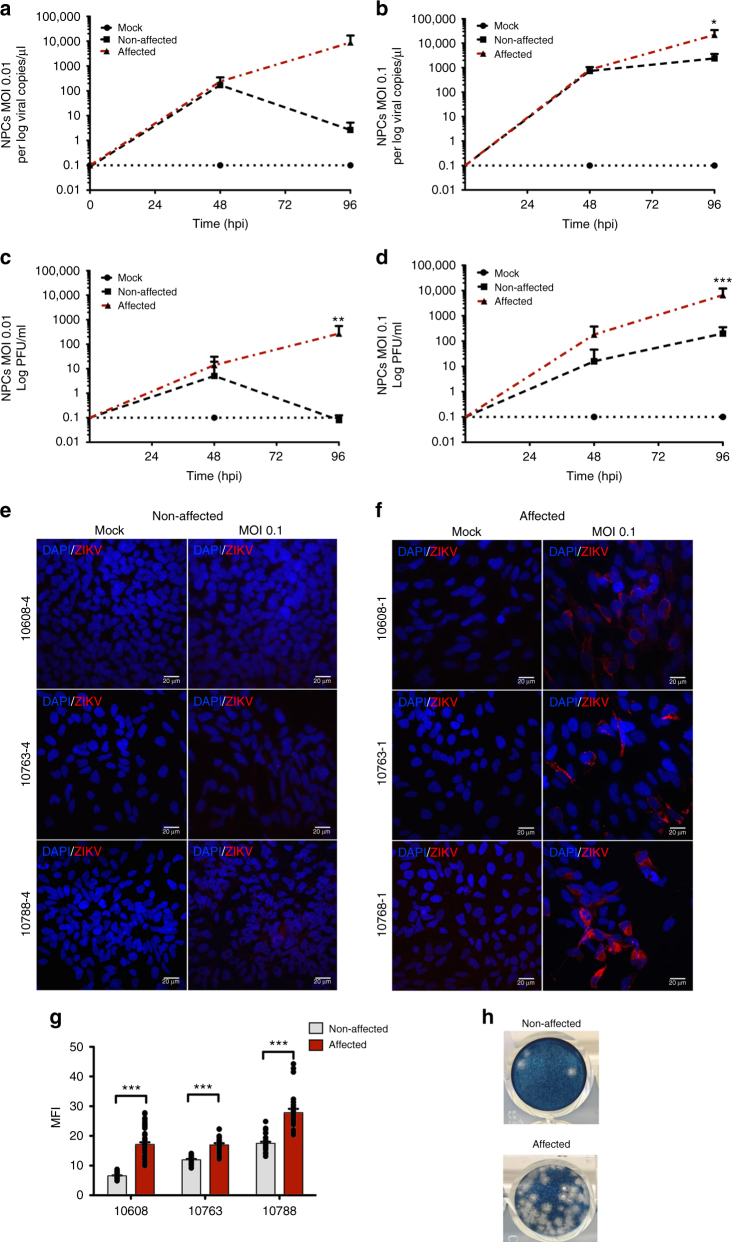
NPCs from affected twins increased susceptibility to ZIKV^BR^ infection compared to the non-affected ones (see also Supplementary Fig. 2). **a**, **b** Viral copies in supernatant of NPCs infected at an MOI = 0.01 (**a**) or MOI 0.1 (**b**) as determined by RT-qPCR. Graphs show mean ± SEM of NPCs from #10608, #10763, and #10788 twins. *p < 0.05, **p < 0.01, and ***p < 0.001 one-way ANOVA with Tukey’s post hoc analysis. **c**, **d** Zika PFU/mL in NPCs supernatant (**c**) MOI = 0.01 and (**d**) MOI = 0.1; #10608, #10763, and #10788 twins; mean ± SEM; **p < 0.01, ***p < 0.001; one-way ANOVA with Tukey’s post hoc analysis. **e**, **f** Zika staining in non-affected (**e**) and affected (**f**) twins at an MOI of 0.1 at 96 hpi; #10608, #10763, and #10788 twins. Scale bar = 20 µm. **g** MFI quantification at 96 hpi (MOI = 0.1; *n* ≅ 20 technical replicates per group; mean ± SEM; ***p < 0.001; Student’s *t* test). **h** Representation of a well stained with ZIKV collected from #10608 NPC supernatant at an MOI of 0.1 at 96 hpi

Next, we investigated the viral load by measuring ZIKV^BR^ copy numbers in NPCs’ culture supernatants and plaque-forming units (PFU) after 48 and 96 hpi. Viral copy number represents total viral genome sequences in supernatant while PFU represents only infective viral particles. Using MOI 0.1, the CZS-affected twins’ NPCs produce significantly more viral copies/μL at 96 hpi (*p* value = 0.0013, one-way ANOVA with Tukey’s post hoc analysis) (Fig. 3a, b, Supplementary Fig. 2). PFU/mL quantification in culture supernatant was significantly higher in the affected twins’ NPCs after 96 hpi (MOI 0.01, *p* value = 0.0024; MOI 0.1, *p* < 0.0001, one-way ANOVA with Tukey’s post hoc analysis) (Fig. 3c, d, h and Supplementary Fig. 2). Confocal analysis shows significantly higher mean fluorescence intensity (MFI) of ZIKV staining in affected as compared to non-affected subjects’ cells (Fig. 3e, f) at 96 hpi (MOI 0.1, *p* value < 0.001, Student’s *t* test) (Fig. 3g). These results demonstrate that NPCs from CZS-affected twins are significantly more susceptible to ZIKV infection.

### RNA-Seq analysis

**Fig. 4:**
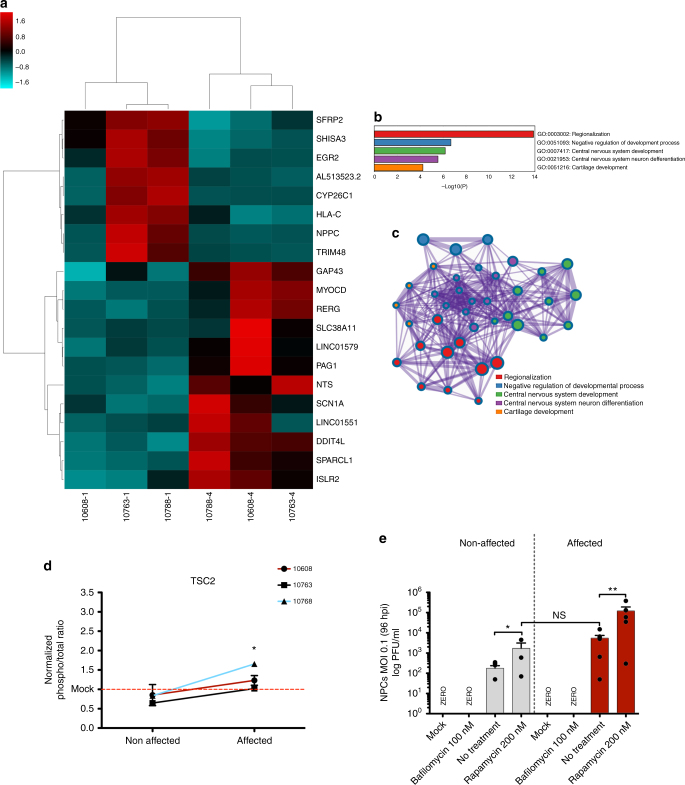
NPC gene expression analyses by RNA-Seq in cultured cells prior to ZIKV infection (see also Supplementary Fig. 3). **a** Heatmap representation and clusterization of top 20 most significant DEGs (*p* < 0.001; edgeR exact test) in NPCs in culture prior to ZIKV infection, in cells derived from non-affected (#10608-4, #10763-4, and #10788-4) and CZS-affected (#10608-1, #10763-1, and #10788-1) twins. Scale bar = *Z* score. **b** Enriched GO terms (*p* value < 0.0001, cumulative hypergeometric distribution). **c** Enriched GO terms interaction network. The thickness of the lines connecting the circles indicates the number of interactions between genes included in each GO term sub-category represented by each circle. **d** TSC2 activity analysis by multiplex array (#10608, #10763, and #10788 twins; mean ± SEM; *p < 0.05; Student's *t* test). **e** PFU/mL measurement in infected NPCs after bafilomycin/rapamycin treatment (#10608, #10763, and #10788 twins; mean ± SEM; *p < 0.05; **p < 0.01; NS = not significant; one-way ANOVA with Tukey’s post hoc analysis)

RNA sequencing (RNA-Seq) analysis of DZ twins-derived NPCs prior to ZIKV infection showed 64 differentially expressed genes (DEGs) (*p* < 0.001, edgeR exact test, Supplementary Fig. 3a and Supplementary Data 3) when the NPCs from the affected and non-affected groups were compared. The gene with the most significant (*p* value = 1.45E−07, corrected *p* value = 0.0058, edgeR exact test, Supplementary Data 3) difference in expression is *DDIT4L*, which is an inhibitor of mTOR signaling. *DDIT4L* showed an average 12.6-fold lower mRNA level in the NPCs from affected twins compared with non-affected (Fig. 4a and Supplementary Data 3). In addition, we found a significant (*p* value < 0.0001, cumulative hypergeometric distribution) enrichment of five different Gene Ontology (GO) terms among the 64 DEGs detected in the RNA-Seq experiment (Fig. 4b). The top most significantly enriched GO is “regionalization,” which includes the related GO cluster members “pattern specification process,” “anterior/posterior pattern specification,” “embryonic morphogenesis,” and “embryo development” (Supplementary Data 4). Among the DEGs belonging to the regionalization GO term are *FOXG1* and *LHX2*, which were down-regulated in affected twins compared with non-affected.

Noteworthy, *FOXG1* has been linked to a wide range of congenital brain disorders^11^ and disrupting mutations of one allele of *FOXG1* results in the core FOXG1syndrome phenotype, which includes postnatal microcephaly and severe intellectual disability^12^. *LHX2* is an early neural marker that regulates neural differentiation by attenuating the Wnt signaling^13^. Here we found that *LHX2* mRNA expression was on average 9.6-fold decreased in the CZS-affected twins’ NPCs compared with non-affected, which is consistent with the observed increase in expression in the NPCs from CZS-affected twins of genes related to Wnt signaling such as *EGR2*, *SFRP2*, and *WNT7A* (Fig. 4a, Supplementary Fig. 3a). The patterns of change in gene expression between CZS-affected and CZS-non-affected twins for all six genes were confirmed by RT-qPCR in the twins’ NPC samples, as seen by a Pearson's correlation *r*^2^ = 0.98 between the fold-changes (FCs) measured by RNA-Seq and RT-qPCR (Supplementary Fig. 3b). Interestingly, no difference in the levels of expression of the six genes was observed between the hiPSCs of affected and non-affected twins from which the NPCs were originated (Supplementary Fig. 3c), indicating that the differences in expression appear after differentiation. It should be noted that the above genes also belong to the “central nervous system development”-enriched GO term (Fig. 4b, see Supplementary Data 4), and members of the enriched GO clusters related to the 64 DEGs exhibit extensive interactions among them as shown in Fig. 4c.

### mTOR pathway analysis

We performed a Cell Signaling Multiplex Assay (Millipore, Billerica, MA) to test for the activity of proteins in the mTOR signaling pathway. NPCs from the CZS-affected group showed increased activity of TSC2 (*p* value = 0.0056, Student’s *t* test) (Fig. 4d). However, other proteins involved in mTOR pathway regulation did not show difference between the two groups (Supplementary Fig. 4). To further test for the role of mTOR signaling in ZIKV infection of NPCs, we used an inhibitor and an activator of the pathway. After infection, cells were treated for 96 h with either 200 nM rapamycin, an mTOR inhibitor, or 100 nM bafilomycin, an indirect mTOR signaling inductor. Bafilomycin completely inhibited viral release while rapamycin increased viral release in non-affected as well as affected twins’ NPCs (Fig. 4e). However, non-affected twins’ NPCs treated with rapamycin showed a similar PFU/mL to that observed in non-treated cells from the affected twins (Fig. 4e) supporting that mTOR individual response is an important factor for successful ZIKV infection.

## Discussion

We were able to study pairs of twins exposed to ZIKV infection during pregnancy, in which at least one of the babies was born with CZS. This is a rare and unique cohort to test whether the host genetic background plays any role in determining CZS outcome. Altogether, five discordant and DZ pairs of twins plus three concordant (one DZ and two MZ) were clinically evaluated and had samples collected. Such proportion of affected vs. non-affected twins led us to suggest a genetic basis for CZS susceptibility. No rare potentially pathogenic variant was assigned in our cohort that could explain susceptibility to CZS, allowing us to exclude monogenic dominant inheritance but not recessive or multifactorial inheritance, due to our relatively limited sample size.

NPCs from affected and non-affected groups had similar growth patterns before ZIKV^BR^ in vitro infection (Fig. 2e). This may suggest that affected babies would not develop microcephaly if not exposed to ZIKV during mothers’ pregnancy. After ZIKV^BR^ infection, NPCs from affected babies showed growth reduction and higher viral replication when compared to cells from non-affected babies.

Therefore, we decided to use RNA-Seq to look for intrinsic differences in NPC gene expression between the two groups, which could be associated with such differential susceptibility to ZIKV^BR^. NPCs from non-affected and CZS-affected twins showed a significantly differential gene expression signature: we found a total of 64 significant DEGs when comparing the NPCs from the three non-affected twins with the three affected ones (Supplementary Data 3). These DEGs belong to five significantly enriched GO terms, all related to regionalization and development (Fig. 4b and Supplementary Data 4). In mammalian development, neurogenesis can be drastically affected by variations in cell proliferation and/or unbalanced neural stem cell differentiation and such interferences can cause microcephaly or megalencephaly^14^. For this reason, in neural stem and progenitor cells, complex and tightly coordinated gene expression programs operate, which control cell cycle and cell growth. Therefore, variants in regulatory regions can deregulate such processes as well as trigger different epigenetic responses. Epigenetic effects involving DNA methylation and histone modifications give rise to differences in gene expression during development, and epigenetic mechanisms seem to allow an organism to respond to the environment through changes in gene expression^15^.

The gene with the most significant difference in the transcriptome analysis was *DDIT4L*, which is an inhibitor of mTOR signaling; *DDIT4L* showed an average 12.6-fold lower mRNA level in the NPCs from affected twins compared with non-affected twins. The literature shows that inhibition of mTORC1 in late corticogenesis^16^ or hyper-stimulation in early corticogenesis in mice causes microcephaly, among other neurological features^10, 17^. Dengue and Chikungunya viruses are known to induce mTORC1 inhibition utilizing a non-canonical pathway to promote cap-dependent viral RNA translation^18–20^. Non-structural proteins (NS4A and NS4B) of ZIKV have been shown to also inhibit mTOR pathway^21^. McGrath et al.^9^ observed two distinct NPC response patterns to ZIKV infection, the interferon and the mTOR pathways. Here we show that only TSC2 activity was significantly higher in affected individuals (Fig. 4d) and that mTOR activity modulation by rapamycin and bafilomycin directly modulates ZIKV replication (Fig. 4e). Although the mechanistic principle of mTOR and Wnt signaling in neurodevelopment and response to flavivirus infection is well established, we suggest that host molecular innate control of such pathways could significantly impact CZS outcome explaining twins’ discordance.

Taken together, these data show that NPCs derived from non-affected and CZS-affected twins have significantly different gene expression signatures of neural development genes and it may contribute to the different susceptibilities to ZIKV^BR^ infection. In short, we compared the impact of ZIKV^BR^ infection in NPCs from three pairs of DZ discordant twins for CZS, demonstrating a highly increased susceptibility to the pathological effects of ZIKV^BR^ infection in the affected as compared to the non-affected group. We could not identify a major *locus* associated with this condition suggesting that CZS may be a multifactorial disorder. Based on our gene expression analyses, we suggest that host variants in regulatory regions leading to different epigenetic responses may be a key parameter to predict the success of ZIKV^BR^ infection in fetuses’ NPCs. Moreover, confirming the influence of the host genome in CZS predisposition sheds light into ZIKV^BR^ molecular pathology and opens a path for drug development targeting specific core pathways to possibly inhibit viral replication and/or ameliorate cellular pathology.

## Methods

### Human subjects

Twins from six Brazilian states (Paraíba, Pernambuco, Sergipe, Bahia, Minas Gerais, and São Paulo) in which at least one was affected by CZS were selected for this study. Five pairs of DZ-D (dichorionic and diamniotic), one pair of DZ concordant and two pairs of MZ concordant twins for microcephaly were examined in this cohort. Zygosity was confirmed by whole-exome sequencing (WES) and by microsatellite analysis (Supplementary Table 1). In addition, we collected saliva samples from 10 unrelated CZS babies (#10720-1, #10725-1, #10727-1, #10729-1, #10739-1, #10661-1, #10693-1, #10701-1, #10741-1, and #10747-1) for WES. All babies were born from mothers negative for previous STORCH infections and affected babies’ head circumference were three standard deviations (SD) below the mean for the given age, sex, and gestation stage at birth^22^. Among the DZ-D twins selected in this study, we were able to collect and isolate peripheral blood mononuclear cell from three pairs only (#10608, #10763, and #10788). Clinical data from the babies and their respective mothers are detailed in Supplementary Data 1. The diagnosis of microcephaly due to ZIKV infection (CZS) in most of all affected subjects selected for this cohort was confirmed by either neuroimaging (Fig. 1g and Supplementary Data 1), serology, or by the mother reporting ZIKV infection symptoms during pregnancy. All babies had saliva samples collected using OraGENE Kit (Oragene^TM^ DNA Collection Kit, DNA Genotek Inc., Ottawa, ON, Canada) and the DNA was extracted following the manufacturer’s’ protocol. All mothers signed informed consents approved by the Human Research Ethics Committee from Biosciences Institute, University of São Paulo (protocol no.: 184/2016).

### Cell lines and maintenance of hiPSCs

hiPSCs were generated from CD71+ cells, which were isolated from three pairs of DZ-D twins’ peripheral blood samples. CD71-positive cells were sorted using magnetic labeled antibody (Miltenyi, Supplementary Table 4) following the manufacturer’s instructions. All hiPSC lines were tested for ZIKV infection by RT-qPCR using primers described in Supplementary Table 2 and the results were negative. The reprogramming protocol was performed with episomal vectors system (pCXLE-hOCT3/4-shP53-F, Addgene plasmid 27077; pCXLE-hSK, Addgene plasmid 27078; pCXLE-hUL, Addgene plasmid 27080; gift from Shinya Yamanaka), according to Okita et al.^23^, and using the Amaxa human CD34+ cells Nucleofection Kit (Lonza), following the manufacturer’s recommendations.

Three days after nucleoporation, cells were seeded on irradiated murine embryonic fibroblasts (Millipore, A24903) in embryonic stem cell medium (Dulbecco's modified Eagle's medium (DMEM)/F12 supplemented with 2 mM GlutaMAX-I, 0.1 mM non-essential amino acids, 100 μM 2-mercaptoethanol, 20% of knockout serum replacement (all provided by Life Technologies), 10 ng/mL of bFGF (Peprotech), 0.25 mM NaB, 0.5 mM VPA, 2 μM thiazovivin, 0.5 μM PD 0325901 and 2 μM SB 431542; all provided by Tocris Bioscience). The typical hiPSC colonies were transferred to hESC-qualified Matrigel (Corning)-coated 60 mm petri dishes (Corning) and cultured in Essential 8 Medium (Gibco) with 100 μg/mL normocin (InvivoGen). All derived cell lines were checked for mycoplasma contamination periodically.

### Serological analysis

IgG antibodies analysis for ZIKV by ELISA was performed in all three mothers’ serum samples using two distinct methods:

*ZIKV ELISA method—first analysis*: Specific IgG antibodies present in serum samples were evaluated by ELISA according to a modified protocol based on Euroimmun ZIKV ELISA^24^. ELISA plates (Corning Inc., New York, NY, USA) were coated with a recombinant fragment of ZIKV NS1 (∆NS1) (100 ng/well) or with equimolar amount of a whole recombinant NS1 protein of DENV2^25^ in a pH 7.2 phosphate-buffered saline (PBS) solution. The recombinant ZIKV proteins were encoded by *Escherichia coli* BL21-Codon Plus DEIII strain (Stratagene, La Jolla, CA, USA) transformed with pET28a plasmids (GenScript, USA) carrying ZIKV ∆NS1 or NS1 sequences derived from the Brazilian strain (GenBank reference number ALU33341). The ∆NS1 corresponded to the last 100 C-terminal amino acids of the NS1 protein, which conferred higher specificity of the detected anti-NS1 antibodies with regard to other flaviviruses. The soluble protein fractions were obtained after hydrostatic pressure lysis (Artepeças, Brazil) in cells suspended in lysis buffer (10 mM of Bis-Tris Propane, 150 mM of NaCl, 1 mM of phenylmethylsulfonyl fluoride and pH 8.5). The recombinant proteins were purified by affinity chromatography using a nickel-containing resin (Hiprep FF 5 mL, GE Healthcare Life Sciences) in a ÄKTA–AVANT device (GE Healthcare Life Sciences). The column was washed with buffers containing 50 and 100 mM imidazole and the bound proteins were eluted from the column with with 500 mM imidazole. Samples containing the recombinant proteins were submitted to a second chromatographic step based on size exclusion chromatography using a Hiprep-SephacrylS-200HR 26/60 column (GE Healthcare Life Sciences). The purified proteins were finally submitted to a final concentration step using nickel affinity chromatography based on the same experimental conditions applied in the first purification step. The purified samples were sorted in 15% polyacrylamide gels and quantified by means of image software (Image Lab, Build 16, Version 4.1, Bio-Rad Laboratories). The purified proteins were stored at −20 °C for subsequent uses. Next, after pre-incubation, the diluted samples were incubated in blocked plates (1.5 min, at room temperature) and the following steps were performed as described elsewhere^26^. The optical density (OD) reading was measured at 492 nm in a plate reader (Labsystems Multiskan, ThermoScientific, Waltham, MA, USA). A signal-to-cut-off was calculated based on control samples and OD value ≥0.76 were regarded as positive.

*ZIKV ELISA method—second analysis*: We used an ELISA Kit for detection of IgG antibodies against ZIKV (Euroimmun, Lübeck, Germany) according to the manufacturer’s instructions. In short, sera were diluted 1:100 in sample buffer and incubated 37 °C for 60 min in a microplate well; a standard curve, positive and negative controls, provided by the manufacturer, were used. The OD was measured in a Multiskan EX (Thermo Scientific) and values ≥1.1 were regarded as positive.

### Computed tomography scan

Two pairs of discordant twins (#10608 and #10763) were submitted to computed tomography scan using 64 Channels Multislice (Philips) with 120 kVp, 131 mA, portion thickness of 2.0, portion space of 1.0, 154 images in series, reconstruction diameter of 197 mm, and matrix of 512 × 512. Neuroimages were analyzed using the ImageJ software.

### Next-generation sequencing and variants filtering

WES was performed on DNA samples from saliva from 8 CZS-affected twins plus 10 unrelated affected babies. All sample libraries were prepared using Agilent’s SureSelect Human All Exon V6 (Agilent Technologies, Santa Clara, CA, USA) and were sequenced on Illumina HiSeq 2500 (Illumina, San Diego, CA, USA) for paired-end reads of approximately 100 × 100 bp. An average on-target coverage of 57.6× was achieved. Base coverage above 10 reads was 94.6%. Alignment of the sequences was performed with the Burrows-Wheeler Aligner (BWA-MEM)^27^. The Picard (http://broadinstitute.github.io/picard/) and Genome Analysis Toolkit (GATK-3.7)^28^ were used for data processing and variant calling with Unified Genotyper tool. Annotation of variants was performed with ANNOVAR^29^ and multiple public databases: the 1000 Genomes Project National Institutes of Health, the 6500 Exome Sequencing Project Washington University, and 609 elderly Brazilian controls from our research center^30^. Our cohort sequencing data was compared with the cohort of 609 Brazilian healthy individuals^30^. We did not identify pathogenic or probably pathogenic statistically significant variants in genes related to Mendelian inherited microcephaly or syndromes associated with microcephaly (Supplementary Data 2), according to ACMG guidelines^31^. We did not find statistically significant variants in genes involved with ZIKV replication in the host cell as potential susceptibility factors for CZS^32–34^ (Supplementary Table 3). Statistical power was calculated using the methods described elsewhere^35^ for case–control discrete trait with the following parameters: number of controls (609); number of affected individuals (18); expected risk allele frequency (0.05); expected disease prevalence (0.06); expected relative risk per risk allele (7:1).

A second analysis consisted of evaluating potential damaging/deleterious variants (missense and loss of function) with population frequency below 5%, which were present in higher frequency in affected babies compared to our Brazilian cohort (SABE609/ABraOM). Variants were also filtered for depth of coverage above 10 reads and allelic balance between 0.3 and 0.7. We applied distinct scores for genes with heterozygotes (score 1) and homozygotes (score 2) variants, selecting those classified as probably deleterious/damaging in at least one of five selected prediction tools (M-CAP, http://bejerano.stanford.edu/mcap/; SIFT, http://sift.jcvi.org/; LRT^36^; MutationTaster, http://www.mutationtaster.org/; and DANN^37^. *χ*^2^ test followed by multiple tests correction (Bonferroni), comparing affected twins with our control cohort was performed for all candidate genes.

### MLPA assay

Total DNA was extracted from cultured cells using the NucleoSpin Tissue Kit (Macherey-Nagel), followithe ng supplier’s instructions. MLPA analysis was performed with subtelomeric kits (P036 and P070; MRC-Holland) to detect chromosomal imbalances, as previously described^38^ (see Fig. 1d).

### Differentiation of human hiPSCs into NPCs

hiPSCs were maintained for 3 days with E8 medium (Life Technologies). On the fourth day, the medium was changed to 0.5x NB medium (DMEM/F12 medium supplemented with 0.5x N2 and 0.5x B27 (all provided by Life Technologies) added with 1 μM dorsomorphin (Tocris) for 48 h. Further, the colonies were detached from the plate and cultured in suspension as embryoid bodies (EBs) for 5 days in ultra-low attachment plates. The EBs were plated on Matrigel-coated plates with NBF medium (DMEM/F12 medium supplemented with 0.5x N2, 0.5x B27, 20 ng/mL fibroblast growth factor 2, 20 ng/mL epidermal growth factor, and 1% penicillin/streptomycin). The emerged rosettes were manually picked, dissociated, and plated in a double-coated plate with poly-ornithine (10 μg/mL, Sigma-Aldrich) and laminin (2.5 μg/L, Gibco). The NPC population was expanded using NBF medium. hiPSCs and NPC validation was performed by RT-qPCR and/or immunofluorescence and flow cytometry using primers and antibodies, respectively, described in Supplementary Tables 2 and 4.

### RNA-Seq assay

Total RNA from NPCs was extracted using the RNeasy Micro Kit (Qiagen, 74004), treated with TURBO DNase (Ambion, AM2238) for 1 h at 37 °C, and then re-purified with the Qiagen RNeasy Micro Kit. RNA samples were quantified using the Qubit RNA HS Assay Kit (Thermo Fisher Scientific, Q32852); purity was evaluated using NanoDrop ND-1000 Spectrophotometer (NanoDrop Technologies) and the integrity was verified using the Agilent RNA 6000 Pico Kit (Agilent Technologies, 5067-1513) in the 2100 Bioanalyzer Instrument (Agilent Technologies). Stranded tagged cDNA libraries were prepared using the KAPA Stranded mRNA-Seq Kit (Illumina, KK8421) and cluster generation was performed using the Illumina HiSeq 4000 PE Cluster Kit (Illumina, PE-410-1001). Tagged libraries were pooled and sequenced (300 cycles, paired-end sequencing) in the Illumina HiSeq 4000 instrument using a HiSeq 4000 SBS Kit (Illumina, FC-410-1003). Raw reads were preprocessed using the standard Illumina pipeline to segregate multiplexed reads. Sequence quality was checked using the FastQC program (http://www.bioinformatics.babraham.ac.uk/projects/fastqc). Data were preprocessed with the read trimming and cropping tool Trimmomatic v.0.30^39^. Sequences were then aligned to the human reference genome GRCh38/hg38 using STAR (v. 2.5.0a)^40^. Mapped reads were counted using the Ensembl GRCh38.83 transcriptome reference through the R software package GenomicRanges^41^, and the statistical analyses of differential expression between samples were done using EdgeR (v. 3.16.5)^42^. Networks of related enriched GO terms were created and analyzed with Metascape^43^.

### RNA-Seq validation by RT-qPCR

Total RNA from hiPSCs and NPCs was extracted as described above. The reverse transcription (RT) reaction was performed with 35 ng of each total RNA sample using the SuperScript IV First-Strand Synthesis System (Life Technologies, cat. #18091050) and random hexamer primers in a 20 μL final volume. The obtained cDNAs were diluted 10 times in water and quantitative PCR was performed using 2.5 μL of each diluted cDNA in a total volume of 10 μL containing 1× LightCycler 480 SYBR Green I Master Mix (Roche Diagnostics, cat. #04707516001) and 800 nM of each primer in a LightCycler 480 System (Roche Diagnostics). Each RT-qPCR was run in three technical replicates and primers are shown in Supplementary Table 2. The *TBP* gene (NM_003194.4) was used as the reference for internal normalization.

### Neurosphere formation

Neuronal progenitor cells were cultured up to 80% confluence and split with Accutase (Gibco, A1110501). Cells were counted and reseeded at confluence of 8 × 10^5^ cell/cm^2^ on poly-ornithine/laminin-coated 60 mm petri dishes. Six hours later the cells were detached after two washes with 1× PBS followed by scraping and transferred to ultra-low attachment 6-well plates and cultured with NBF medium at 37 °C, 5% CO_2_ (g), and 95 r.p.m. (0.1009 g) agitation until the end of the experiment. The pictures were acquired using EVOS Cell Imaging System (Thermo Fisher Scientific). A total of 10 pictures per group were analyzed using the ImageJ software. This experiment was performed twice.

### *In situ* immunofluorescence and flow cytometry staining

hiPSCs and NPCs cultures were fixed with 4% PFA followed by permeabilization with 0.01% Triton X-100 and then blocked with 5% bovine serum albumin (BSA) in 1× PBS (1 h). After that, the cells were incubated overnight with primary antibodies (Supplementary Table 4) at 4 °C and subsequently incubated with secondary fluorescent antibodies for 1 h at room temperature. The final step was the DAPI (4',6-diamidino-2-phenylindole) staining for 2 min at room temperature. For ZIKV immunofluorescence stain, cells were fixed with ice-cold acetone for 20 min, air dried, blocked with 5% BSA, and stained for 1 h at 37 °C in a high humidity chamber with primary antibody (Supplementary Table 4). Subsequently, secondary stain protocol followed as described above. Confocal analysis was performed using Zeiss LSM 800. In order to analyze the MFI of ZIKV infection, approximately 50 cells per group were measured for the respective fluorescent channel. This experiment was performed in duplicate. Flow cytometry staining for neural progenitor marker (Nestin; Musashi-1) were performed as follows. A total of 1 × 10^6^ cells were fixed for 20 min at room temperature using Fix and Perm Kit (Invitrogen) solution A. The cells were washed with 1× PBS and permeabilized using the Fix and Perm Kit solution B, incubated with primary antibody or isotype control antibody for 1 h on ice. After washing with 1× PBS, cells were permeabilized and incubated with secondary antibody for 1 h on ice. Flow cytometry data acquisition was performed using FACS Aria (BD) collecting 10,000 events/run. Analysis was carried using the FlowJo software.

### Zika virus

ZIKV^BR^ was a courtesy of Dr. Pedro Vasconcelos, InstitutoEvandro Chagas, Brazil^44^. Viral stock was established after viral propagation for two serial passages in VERO cells (ATCC^®^ CCL-81™) in serum-free medium (VP SFM, Thermo scientific).

### Measurement of viral burden

Supernatants of cell culture were collected and used for ZIKV titration by both, plaque assay, and absolute RT-qPCR. For plaque assay, an amount of 6 × 10^4^ VERO cells/well were seeded in 24-well plates 48 h before the assay. Samples were serially diluted in DMEM culture medium from 10^−1^ to 10^−6^, applied in duplicates of 200 µL to each well, and incubated for 30 min at 37 °C. After virus adsorption, wells were overlaid with culture medium containing carboxymethyl cellulose (1%) and incubated at 37 °C. After 5 days, plates were drained, washed with PBS, and stained with 0.1% naphthol blue-black, 1.6% sodium acetate in 6% glacial acid acetic for 30 min. Plaque formation units were visually determined in the most appropriate viral dilution and expressed as PFU/mL. ZIKV RNA obtained from samples’ supernatants was extracted using Viral RNA Mini Kit (Qiagen), according to the manufacturer’s protocol and viral RNA copies were calculated by RT-qPCR.

For absolute RT-qPCR titration, a standard curve was generated with a double-strand DNA fragment (KU365779.1:1179-1255) with sequence corresponding to a region of Zika E protein (Supplementary Table 2). The fragment was quantified by fluorometry (QuBit, Thermo Fisher) and the concentration required for a specific number of target sequences was calculated. Six dilutions were performed in TE + EGTA 1 mM buffer, generating the standard dilutions containing 6 × 10^7^, 6 × 10^6^, 6 × 10^5^, 6 × 10^4^, 6 × 10^3^, or 6 × 10^2^ copies at each 3 µL. cDNA samples were obtained after reverse transcription of Zika RNA using primers described elsewhere^45^ and the SuperScript III Reverse Transcriptase Kit (Thermo Fisher). Samples were amplified along with standard curve dilutions, in three replicates each. Mean Cts were applied to standard curve equation to determine cDNA copy numbers in each sample. The total Zika RNA copy numbers were calculated by multiplication of the cDNA copy number by a conversion factor, which considered all dilutions made during the RNA extraction to qPCR protocol.

### Infection of the NPCs and neurospheres with ZIKV^BR^

NPCs were seeded into 6-well or 24-well plates (Corning) and 2-well chamber slides (Nunc; Thermo Fisher Scientific) to a confluence of 3 × 10^4^ cells/cm^2^. NPCs (monolayer) as well as 3D (neurospheres) were exposed to ZIKV^BR^ (MOI: 0.01, 0.1, and Mock). Monolayer cells were exposed to the virus for 1 h at 37 °C and 5% CO_2_ (g), washed with medium 0.5x NB, and then maintained by up to 96 h (end point). The same procedure was performed with the neurospheres, but with viral exposure for 2 h to ensure complete sphere infection. mTOR inhibition and activation assay was performed by infecting the NPCs (MOI 0.1) as described above, cells were kept in culture with 200 nM of rapamycin (mTOR inhibitor) or 100 nM of bafilomycin (mTOR activator) during the whole infection experiment. Then, ZIKV^BR^ PFU and copy numbers were analyzed from supernatant media, as previously described.

### Cell counting and neurosphere diameter

Quantification of number of cells (for each condition during 4 days) and alterations in neurosphere diameter over time (24 and 96 hpi) were performed with NIH-ImageJ software. Data were determined by ImageJ under original magnification x20 and reported as total number of cells and neurosphere diameter. A total of 10 pictures per group were analyzed.

### mTOR pathway multiplex assay

Cell culture samples were prepared for phosphoprotein and total protein assays using the premixed Magnetic MILLIPLEX MAP 11-Plex Akt/mTOR Phosphoprotein Panel and the Magnetic MILLIPLEX MAP 11-Plex Akt/mTOR total protein Pathway Signaling Kit (cat. # 48-611MAG; 48-612MAG, Millipore) according to the manufacturer’s instructions. In brief, 12.5 µg of protein of each sample was carried out using the manufacturer’s Assay Buffer 2. Then, 25 μl of each standard and the samples were incubated with the target capturing beads on a 96-well plate for 20 h at 4 °C, followed by a 1 h incubation with the appropriate 1x MILLIPLEX MAP Detection Antibody at room temperature. After the washing procedure, 1x MILLIPLEX MAP Streptavidin-PE was added to each well and incubated for 30 min. Without discarding the streptavidin-PE, we added amplification buffer and incubated it for 15 min at room temperature. Samples were washed and re-suspended in assay buffer and the fluorescence was measured using a Luminex MAGPIX (Luminex, Austin, TX, USA) for magnetic assays. Each sample was analyzed in triplicate.

### Quantification and statistical analysis

Two-tailed unpaired *t* test was used for pairwise comparison. One-way and two-way ANOVA was performed, followed by post hoc pairwise comparison. Graphpad Prism software was used to perform all statistical analysis (version 6.0). Quantification of data are represented as mean ± SEM and *p* value threshold was * 0.05, ** 0.01, and *** 0.001.

### Data availability

The RNA-Seq data that support the findings of this study have been deposited in the Gene Expression Omnibus NCBI repository under Accession number GSE102128. All other data supporting the findings of this study are available within the article and its Supplementary Information files, or are available from the authors upon request. Due to privacy and consent restrictions, individualized WES data files cannot be uploaded publicly to NCBI, but might be available to researchers upon request and signature of a Data Use Agreement.

## Electronic supplementary material

Supplementary Information

Description of Additional Supplementary Files

Supplementary Data 1

Supplementary Data 2

Supplementary Data 3

Supplementary Data 4
