# Publisher Correction: Discordant congenital Zika syndrome twins show differential in vitro viral susceptibility of neural progenitor cells

**DOI:** 10.1038/s41467-018-03497-1

**Published:** 2018-03-13

**Authors:** Luiz Carlos Caires-Júnior, Ernesto Goulart, Uirá Souto Melo, Bruno Henrique Silva Araujo, Lucas Alvizi, Alessandra Soares-Schanoski, Danyllo Felipe de Oliveira, Gerson Shigeru Kobayashi, Karina Griesi-Oliveira, Camila Manso Musso, Murilo Sena Amaral, Lucas Ferreira daSilva, Renato Mancini Astray, Sandra Fernanda Suárez-Patiño, Daniella Cristina Ventini, Sérgio Gomes da Silva, Guilherme Lopes Yamamoto, Suzana Ezquina, Michel Satya Naslavsky, Kayque Alves Telles-Silva, Karina Weinmann, Vanessa van der Linden, Helio van der Linden, João Ricardo Mendes de Oliveira, Nivia Maria Rodrigues Arrais, Adriana Melo, Thalita Figueiredo, Silvana Santos, Joanna Goes Castro Meira, Saulo Duarte Passos, Roque Pacheco de Almeida, Ana Jovina Barreto Bispo, Esper Abrão Cavalheiro, Jorge Kalil, Edécio Cunha-Neto, Helder Nakaya, Robert Andreata-Santos, Luis Carlos de Souza Ferreira, Sergio Verjovski-Almeida, Paulo Lee Ho, Maria Rita Passos-Bueno, Mayana Zatz

**Affiliations:** 1 Department of Genetics and Evolutionary Biology, Human Genome and Stem Cell Research Center, Biosciences Institute, University of São Paulo (USP), São Paulo – SP, 05508-900 Brazil; 2 Brazilian Biosciences National Laboratory (LNBio), Brazilian Center for Research in Energy and Materials (CNPEM), Campinas – SP, 13083-970 Brazil; 3 Neuroscience laboratory, Department of Neurology and Neurosurgery, Federal University of São Paulo—UNIFESP/EPM, São Paulo – SP, 04039-002 Brazil; 4 Butantan Institute, São Paulo – SP, 05503-900 Brazil; 5 Albert Einstein Hospital, São Paulo – SP, 05652-900 Brazil; 6 Department of Biochemistry, Institute of Chemistry, University of São Paulo (USP), São Paulo – SP, 05508-900 Brazil; 7 Universidade de Mogi das Cruzes, Mogi das Cruzes – SP, 08780-911 Brazil; 8 AACD, Recife – PE, 50080-810 Brazil; 9 Rehabilitation Center—Dr. Henrique Santillo, Goiânia – GO, 74653-230 Brazil; 10 Neuropsychiatry Department and KeizoAsami Laboratory, Federal University of Pernambuco (UFPE), Recife – PE, 50670-901 Brazil; 11 Department of Pediatrics, Federal University of Rio Grande do Norte (UFRN), Natal– RN, 59010-180 Brazil; 12 ISEA, Campina Grande– PB, 58400-220 Brazil; 13 Department of Biology, Paraíba State University (UEPB), Campina Grande – PB, 58429-500 Brazil; 14 Federal University of Bahia (UFBA), Salvador – BA, 40170-115 Brazil; 15 Infectious pediatric laboratory, Medicine School of Jundiaí, Jundiaí – SP, 40170-115 Brazil; 16 Division of Immunology and Molecular Biology Laboratory, Federal University of Sergipe (UFS), Aracaju – SP, 49100-000 Brazil; 17 Heart Institute, Faculty of Medicine, University of São Paulo (USP), São Paulo – SP, 05403-900 Brazil; 18 Department of Clinical and Toxicological Analyses, School of Pharmaceutical Sciences, University of São Paulo (USP), São Paulo – SP, 05508-900 Brazil; 19 Vaccine Development Laboratory, Department of Microbiology, Institute of Biomedical Science, University of São Paulo (USP), São Paulo – SP, 05508-900 Brazil

Correction to: *Nature Communications* 10.1038/s41467-017-02790-9; published online 02 February 2018

The original PDF version of this Article contained errors in the spelling of Luiz Carlos Caires-Júnior, Uirá Souto Melo, Bruno Henrique Silva Araujo, Alessandra Soares-Schanoski, Murilo Sena Amaral, Kayque Alves Telles-Silva, Vanessa van der Linden, Helio van der Linden, João Ricardo Mendes de Oliveira, Nivia Maria Rodrigues Arrais, Joanna Goes Castro Meira, Ana Jovina Barreto Bispo, Esper Abrão Cavalheiro, and Robert Andreata-Santos, which were incorrectly given as Luiz Carlos de Caires Jr., UiráSouto Melo, Bruno Silva Henrique Araujo, Alessandra Soares Schanoski, MuriloSena Amaral, Kayque Telles Alves Silva, Vanessa Van der Linden, Helio Van der Linden, João Mendes Ricardo de Oliveira, Nivia Rodrigues Maria Arrais, Joanna Castro Goes Meira, Ana JovinaBarreto Bispo, EsperAbrão Cavalheiro, and Robert Andreata Santos. Furthermore, in both the PDF and HTML versions of the Article, the top panel of Fig. 3e was incorrectly labeled ‘10608-1’ and should have been ‘10608-4’, and financial support from CAPES and DECIT-MS was inadvertently omitted from the Acknowledgements section. These errors have now been corrected in both the PDF and HTML versions of the Article.

