# Supplementary material for: Discordant congenital Zika syndrome twins show differential in vitro viral susceptibility of neural progenitor cells
Source: Nat Commun. 2018 Feb 2;9:475. doi: 10.1038/s41467-017-02790-9 (PMC5797251; doi:10.1038/s41467-017-02790-9)
Supplement: Supplementary file 1 — Supplementary Information [file 41467_2017_2790_MOESM1_ESM.pdf]

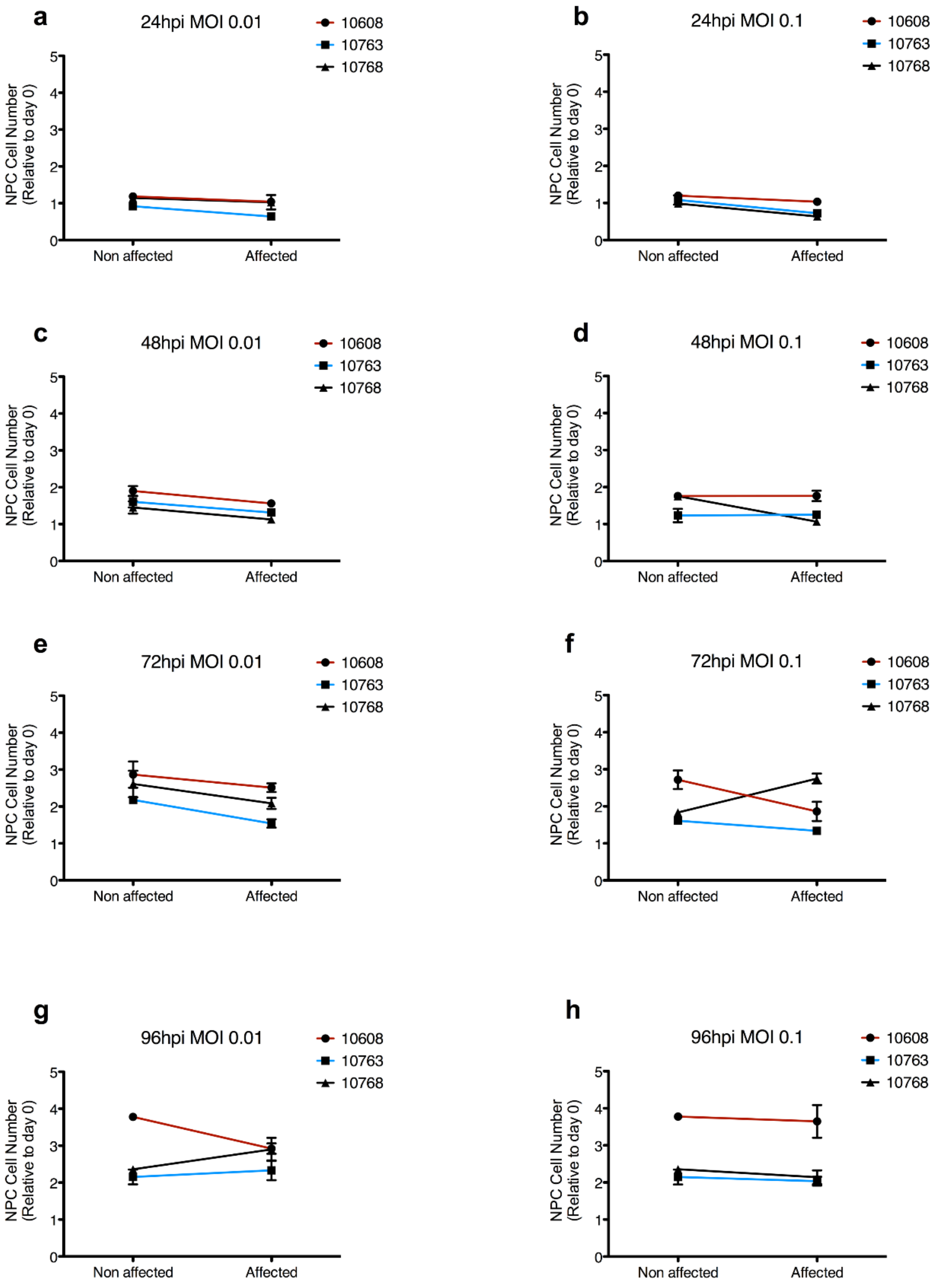

Supplementary Fig. 1 Related to Fig. 2 Cell growth in NPCs after ZIKV^{BR} infection. **a, c, e** and **g** Analysis of cell number of NPCs infected with MOI 0.01 at each time point after ZIKV^{BR} infection (24, 48, 72 and 96 hpi; #10608, #10763 and #10788 twins, mean ± SEM; n = 3 technical replicates). **b, d, f** and **h** Analysis of cell number of NPCs infected with MOI 0.1 at each time point after ZIKV^{BR} infection (24, 48, 72 and 96 hpi; #10608, #10763 and #10788 twins, mean ± SEM; n = 3 technical replicates).

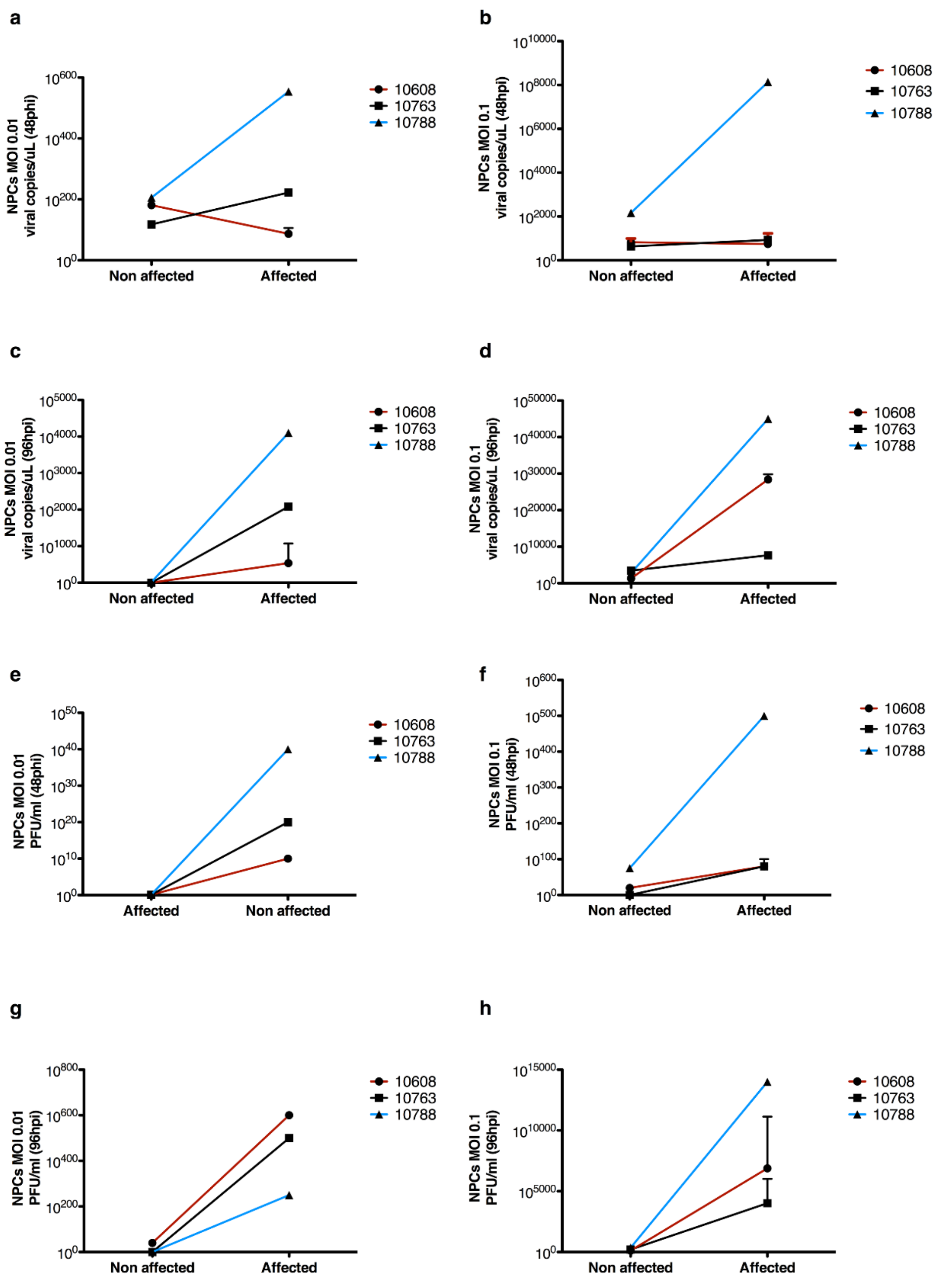

Supplementary Fig. 2 Related to Fig. 3. Twin pairs' difference between quantitative measure of Zika viral copies and PFU/mL. **a-d** Bar graph of Zika viral copies of non-affected and affected babies at each MOI (0.01 and 0.1) after ZIKV^{BR} infection at 48 and 96 hpi (#10608, #10763 and #10788 twins). **e-f** Bar graph of PFU/ml of non-affected and affected babies at each MOI (0.01 and 0.1) after ZIKV^{BR} infection at 48 and 96 hpi (#10608, #10763 and #10788 twins).

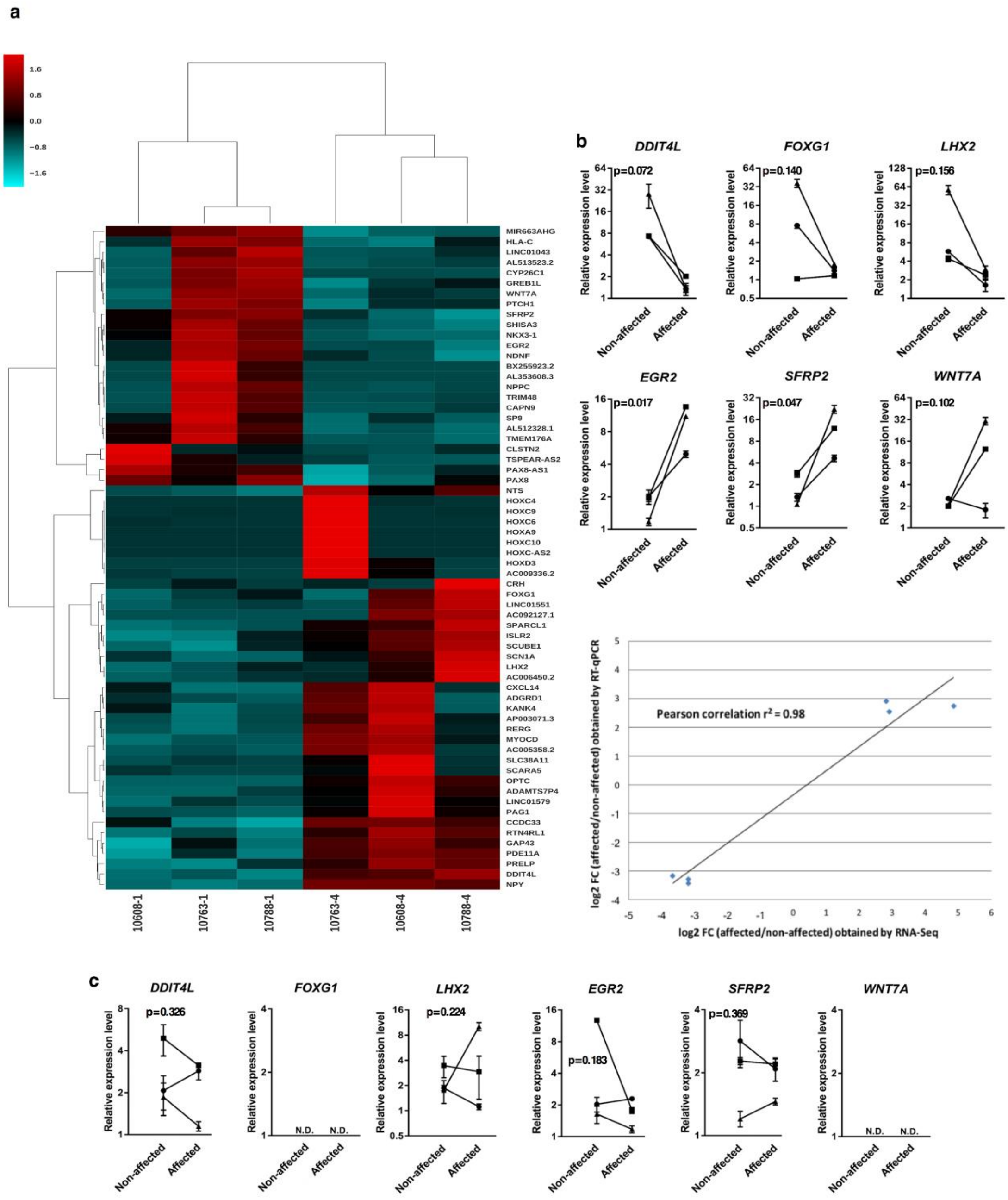

Supplementary Fig. 3 Related to Fig. 4. a Heat-map representation and clusterization of all 64 DEGs ($p < 0.001$, edgeR exact test) in NPCs in culture prior to ZIKV infection, in cells derived from non-affected (#10608-4, #10763-4 and #10788-4) and CZS-affected (#10608-1, #10763-1 and #10788-1) twins. Scale bar = Z-score. **b** Expression measured by RT-qPCR of a set of six selected DEGs in the non-infected NPCs from the non-affected and affected twins from each family, which are connected by solid lines; mean ± SEM; n = 3 technical replicates. The lower panel in (b) shows the correlation

between the fold changes (FC) for NPCs of affected and non-affected twins for the six selected DEGs (each of six points), from data obtained by RT-qPCR (y-axis) and by RNA-Seq (x-axis); a Pearson correlation coefficient r^2 = 0.98 was calculated. **c** Expression measured by RT-qPCR of the set of six selected genes in the hiPSCs from the non-affected and affected twins, before these iPSCs were differentiated to NPCs. Data points from twins of the same family are connected by solid lines. N. D. = not detected. Twins from each family are represented with a different symbol: circles, #10608 twins; squares, #10763 twins; triangles, #10788 twins. (mean ± SEM; n = 3 technical replicates).

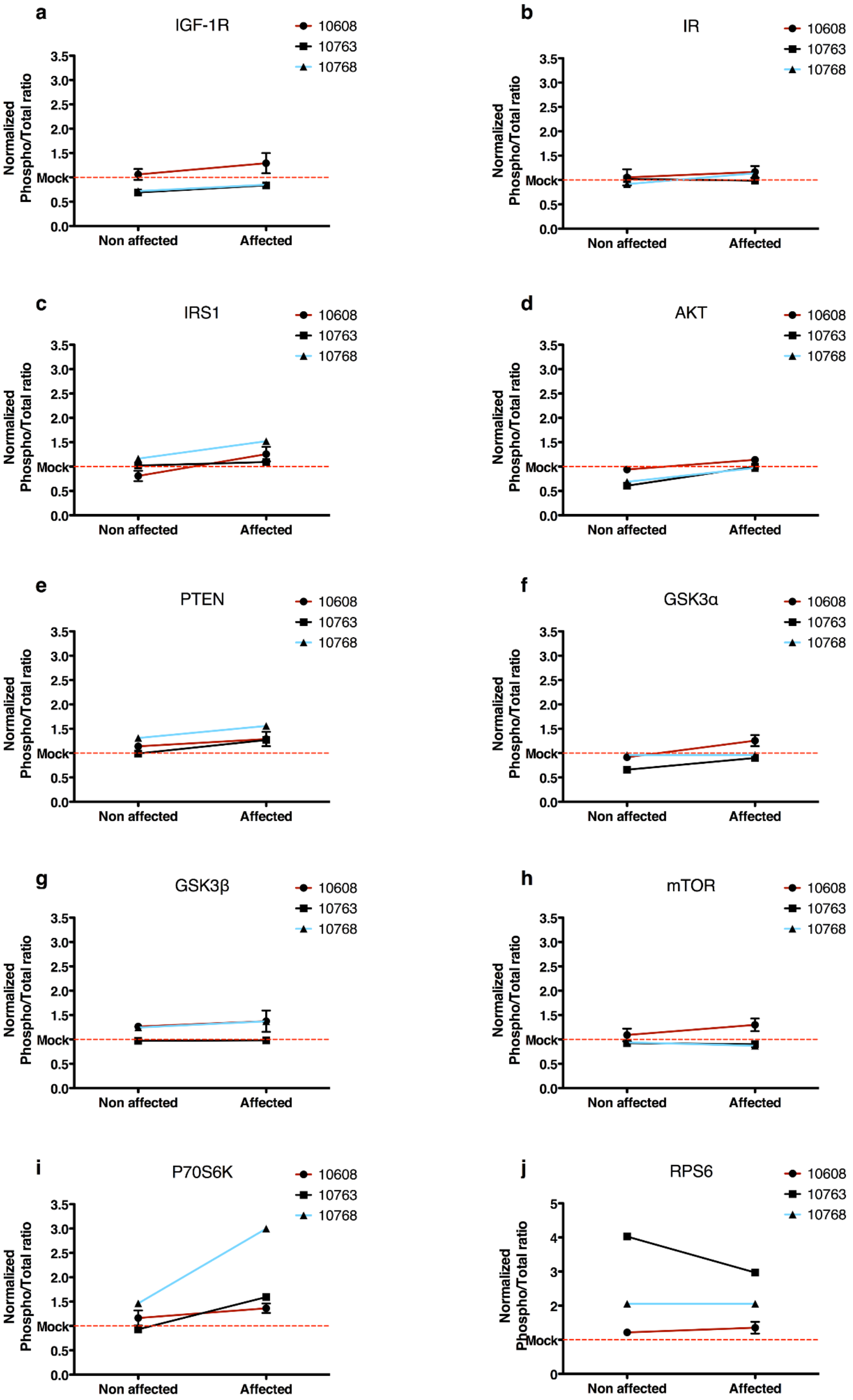
a
IGF-1R
b
IR
c
IRS1
d
AKT
e
PTEN
f
GSK3α
g
GSK3β
h
mTOR
i
P70S6K
j
RPS6
10608
10763
10768
Normalized Phospho/Total ratio
Mock
Non affected
Affected

Supplementary Fig. 4 Related to Fig. 4. mTOR pathway response at 96 hpi. **a-h** Bar graph showing the phosphorylation ratio of IGF1-R, IR, IRS1, AKT, PTEN, GSK3α, GSK3β, mTOR, P70S6K and RPS6 between the NPCs from affected and non-affected babies after $ZIKV^{BR}$ infection. These were determined by Akt/mTOR total protein and phosphoprotein pathway signaling kit (Millipore). (MOI 0.1 - 96 hpi; #10608, #10763 and #10788 twins; n = 3 technical replicates; mean ± SEM; student t-test, each data were normalized by their respective mock).

**Molecular Marker**	**F10789-1**		**F10789-4**	
D4S413	288	295	288	295
D4S415	289	291	289	291
D4S426	162	175	162	175
D4S1535	257	265	257	265
DS41597	97	99	97	99
D12S351	161	163	161	163
D13S156	283	290	283	290
D15S205	159	172	159	172
D16S415	231	231	231	231
D17S1852	293	303	293	303
D18S478	249	249	249	249
D19S220	280	284	280	284
D20S173	127	174	127	174
D21S266	162	169	162	169
D22S315	183	199	189	199

Supplementary Table 1. Microsatellites confirming zygosity

Genes	Sense	Sequence (5'-3')
OCT3/4	Forward	TCCCATGCATTCAAACTGAGG
	Reverse	CCAAAAACCCTGGCACAAACT
NANOG	Forward	TGGACACTGGCTGAATCCTTC
	Reverse	CGTTGATTAGGCTCCAACCAT
ZIKVBR	Forward	CCGCTGCCCAACACAAG
	Reverse	CCACTAACGTTCTTTTGCAGACAT
EGR2	Forward	CTTTGACCAGATGAACGGAGT
	Reverse	AGCAAAGCTGCTGGGATATG
SFRP2	Forward	CGACATAATGGAAACGCTTTG
	Reverse	ATGGTCTTGCTCTTGGTCTCC
WNT7A	Forward	CATGAACTTGCACAACAACG
	Reverse	GAAACTGTGGCAGTGTGGTC
DDIT4L	Forward	GCAGTTTGAGCAGCAAGAAC
	Reverse	GAGGTTGGGTTCAGGAACAA
FOXG1	Forward	CGAGAAGAAGAACGGCAAGT
	Reverse	GAACTCGTAGATGCCGTTGAG
LHX2	Forward	CCAAGGACTTGAAGCAGCTC
	Reverse	GTAAGAGGTTGCGCCTGAACT
TBP	Forward	GAGAGTTCTGGGATTGTACCG
	Reverse	ATCCTCATGATTACCGCAGC

Supplementary Table 2. Primers used in RT-qPCR experiments

**Savidis et al., 2016**	**Zhang et al., 2016**	**Marceau et al., 2016**
AXL	*EMC4*	*STT3A*
EMC1	*EMC6*	*STT3B*
EMC2	*HSPA13*	*RPN1*
EMC3	*OST4*	*SSR2*
EMC4	*OSTC*	*SSR3*
EMC5	*SEC61B*	*AUP1*
EXT1	*SEC63*	*SEL1L*
EXTL3	*SEL1L*	*UBE2J1*
HSA-MIR-451A	*SERP1*	*ASCC2*
HSA-MIR-451B	*SPCS1*	*RPS25*
RAB5C	*SPCS3*	
RABGEF1	*STT3A*	
SSR2		
SSR3		
STT3A		
WDR7		
ZFYVE20		

Supplementary Table 3. Candidate genes described in three functional studies associated with ZIKV replication.

Primary Immunofluorescence Antibodies				
**Cell Type**	**Antibody**	**Host**	**Catalog number**	**Assay dilution**
hiPSC	SSEA4	mouse	ab16287	1.5:100
	OCT3/4	rabbit	ab19857	1:100
NPC	Alexa Fluor 647- anti Nestin	mouse	560393	1:100
NPC	Nestin	rabbit	ab27952	1:200
NPC	Musashi-1	rabbit	ab52865	1:100
ZIKV	D1-4G2-4-15	mouse	mab10216	1:100
Erythroblast	CD71-microbead	mouse	130-046-201	1:25
**Secondary Immunofluorescence Antibodies**				
**Cell Type**	**Antibody**	**Host**	**Catalog number**	**Assay dilution**
hiPSC	Goat-anti-mouse Alexa 488	mouse	A11001	1:1000
	Goat-anti-rabbit CY3	rabbit	A10520	1:1000
NPC	Goat-anti-rabbit CY3	rabbit	A10520	1:1000
ZIKV	Goat-anti-mouse Alexa 488	mouse	A11001	1:1000
NPC	Anti-rabbit HRP	rabbit	7074S	1:2000

Supplementary Table 4. Flow Cytometry and Immunofluorescence antibodies
