# Supplementary material for: Discordant congenital Zika syndrome twins show differential in vitro viral susceptibility of neural progenitor cells
Source: Nat Commun. 2018 Feb 2;9:475. doi: 10.1038/s41467-017-02790-9 (PMC5797251; doi:10.1038/s41467-017-02790-9)
Supplement: Supplementary file 2 — Description of Additional Supplementary Files [file 41467_2017_2790_MOESM2_ESM.pdf]

File Name: Supplementary Data 1
Description: Clinical and serological data from 21 affected and 5 non-affected babies of this study.

File Name: Supplementary Data 2
Description: Genes previously associated with Mendelian microcephaly.

File Name: Supplementary Data 3
Description: List of differentially expressed genes detected by RNA-Seq of NPCs derived from non-affected and CZS-affected twins.

File Name: Supplementary Data 4
Description: Enriched GO terms among the 64 differentially expressed genes between NPCs derived from CZS-affected twins and NPCs from non-affected twins.
